# Influence of Sunlight on Vitamin D and Health Status in Green (*Chelonia mydas*) Sea Turtles with Fibropapillomatosis

**DOI:** 10.3390/ani12040488

**Published:** 2022-02-16

**Authors:** Victoria E. Garefino, Sarah L. Milton

**Affiliations:** Department of Biological Sciences, Florida Atlantic University, 777 Glades Road, Boca Raton, FL 33431, USA; vgarefino2017@fau.edu

**Keywords:** sea turtles, tumor, calcium, parathyroid hormone, UV light, rehabilitation, pathology

## Abstract

**Simple Summary:**

Green sea turtles are an endangered species prone to a disease called fibropapillomatosis (FP). FP causes the growth of large debilitating tumors on the skin, eyes, and shell of sea turtles. Sea turtle rehabilitation facilities often treat turtles with this disease by removing the tumors, but many of these individuals do not survive or the tumors regrow. A way to improve the treatment of these turtles could help the population as a whole. The aim of this study was to compare plasma vitamin D levels in green sea turtles with and without evident FP tumors, as vitamin D influences immune function and overall health. We determined that exposure to more sunlight influences plasma vitamin D levels in sea turtles brought into a rehabilitation facility. We found that tumored individuals arriving at the facility had lower vitamin D and ionized calcium levels and higher parathyroid hormone levels compared to both wild-caught and rehabilitation turtles without evident tumors. Individuals housed in tanks exposed to greater ultraviolet (UV) (sun)light showed greater increases in plasma vitamin D levels and a more successful recovery. The results suggest that increasing sun exposure in rehabilitation facilities may enhance health and recovery in green turtles with FP.

**Abstract:**

Green sea turtles (*Chelonia mydas*) are an endangered species, which as juveniles are prone to the debilitating disease green turtle fibropapillomatosis (FP). Previous work has shown an association between reduced immune function and FP. As vitamin D has been linked to immune function in numerous animals, the aim of this study was to compare vitamin D levels in green sea turtles with and without evident FP and determine if exposure to sunlight would influence vitamin D levels and other health parameters. Various health markers, including vitamin D, in turtles with and without evident tumors being treated at a rehabilitation facility in southeast Florida were compared to apparently healthy wild-caught juvenile green turtles. Turtles receiving treatment were housed in tanks exposed to higher or lower levels of sunlight for up to 6 months. Upon intake, tumored individuals had lower plasma vitamin D and ionized calcium levels and higher parathyroid hormone levels when compared to both wild-caught and rehabilitation turtles without evident tumors. Individuals exposed to greater sunlight showed greater increases in plasma vitamin D and a more successful recovery. The results suggest that increasing sun exposure in rehabilitation facilities may enhance health and recovery in green turtles with FP.

## 1. Introduction

Green turtle fibropapillomatosis (FP) is a debilitating disease that affects sea turtles worldwide. Although it primarily affects juvenile green sea turtles (*Chelonia mydas*), it has been confirmed in all species of hard-shelled marine turtles [1,2,3,4,5,6]. FP is characterized by benign fibroepithelial tumors that can be present on the eyes, skin, shell, and mouth cavity as well as internally [7]. While the disease itself is not considered fatal, morbidity and mortality are high due to tumor growth that interferes with vision, swimming and feeding, and/or internal organ function [8]. The disease is associated with a herpes virus known as Chelonian alphaherpesvirus 5 (ChHV5) [9], though healthy turtles can carry the virus and show no evidence of disease [10]. The expression of the disease is prevalent in polluted environments [11,12,13,14], and FP has also been associated with a suppressed immune system as either a causal or resultant effect [15,16,17,18,19]. The primary current treatment for FP is tumor removal via CO_2_ laser surgery, which is effective but also often results in regrowth of the tumors [20]. Due to the severity of the disease, approximately 75–77% of turtles in rehabilitation facilities with FP either die or are euthanized [21,22]. Finding a way to improve the health of animals being treated for FP may result in better outcomes; increasing vitamin D levels could be one way of doing so.

Vitamin D (vit D) is an essential nutrient in vertebrates that plays many physiological roles. It can be obtained from either dietary sources or be manufactured from precursors generated in the skin upon exposure to ultraviolet (UV) rays [23,24,25]. While vit D is best known for its roles in bone mineralization and calcium homeostasis [26], as a hormone it has been found to have other important functions, including antiproliferative effects on cancer cells, reviewed in [27]; in mice with mammary cancer, vit D was shown to inhibit tumor growth [28]. Vitamin D deficiency has also been associated with a number of pathological conditions including infections [29], autoimmune diseases, and allergies, with major roles in both the adaptive and innate immune systems reported in multiple species [30,31]. In humans, the active form of vit D can increase release of the antimicrobial polypeptide cathelicidin, which acts as a natural defense in many living organisms [32] and has been shown to inhibit vaccinia virus, herpes simplex virus type 1, and retrovirus replication [23]. Links between vit D and immune function have also been reported in non-mammalian vertebrates, such as the European sea bass (*Dicentrarchus labrax*), where fish administered high levels of vit D had greater immune function and phagocytic capabilities than a non-supplemented group [33], and vitamin D3 administration increased cathelicidin release in salmon [34]. As vit D has been shown to benefit the recovery of various species exposed to disease, it is possible that vit D could also improve the recovery of green sea turtles (*Chelonia mydas*) with FP, via improved overall health and/or direct impacts on immune function. 

Vit D is generally obtained through food that is rich in vit D or through UV radiation, such as sun exposure [25]. Many vertebrate species rely on sun exposure for health, as not all diets are rich in vit D, and studies have shown that supplemental UV radiation can help boost vit D levels in reptiles [35], including corn snakes (*Elaphe guttata*) [35] and red-eared sliders (*Trachemys scripta elegans*) [36]. Agamid and iguanid lizards exposed to natural sunlight also had significantly higher levels of vit D than those exposed only to artificial UV light and/or dietary vit D [37]. In a study on juvenile bearded dragons (*Pogona vitticeps*), ultraviolet-B radiation (UVB) exposure resulted in vitamin D levels 5–18 times higher than in the group that received dietary vit D supplementation only [38], while Purgley et al. reported that vitamin D levels of captive green sea turtles kept indoors without any exposure to ultraviolet radiation had lower vitamin D levels than those kept in outdoor facilities [39]. In addition, some studies have shown that vit D levels are lower in rehabilitated turtles than in wild-caught individuals [40,41]. Bloodgood et al. looked at FP as a potential variable related to differing vitamin D levels between individuals, but the interaction was not significant [40]. This study was thus designed to increase our knowledge of the potential link between vitamin D levels in individuals and FP. 

As many rehabilitation facilities have enclosures that limit patient exposure to natural UV light, the objectives of this study were to determine (1) if there are variations in vitamin D levels between turtles brought in to rehabilitation facilities and wild-caught individuals, and (2) if higher levels of sun exposure increase vit D levels in sea turtles undergoing treatment in a rehabilitation facility compared to turtles receiving less UV. Opportunistically, we also looked to see if increased exposure to UV light resulted in reduced regrowth of tumors in animals treated for FP, as higher vit D levels can improve overall health and immune function. We hypothesized that (1) vit D levels would vary between turtles with apparent FP and those turtles without evident tumors, and (2) vit D levels would be higher in turtles exposed to greater levels of sunlight. The results of this work may provide rehabilitation facilities with a mechanism to improve health outcomes in animals with FP.

## 2. Materials and Methods

This study was conducted in collaboration with rehabilitation staff at Gumbo Limbo Nature Center (GLNC: Florida Marine Turtle Permit (MTP) #084), with the researchers at Inwater Research Group (IRG, Marine Turtle Permit #125), and under Marine Turtle Permit #053. The use of animal products was approved by the FAU Institutional Animal Care and Use Committee, protocol number A(T) 18-02, approved 6 April 2018.

To test the hypotheses, we compared vitamin D levels and blood chemistry values in turtles without evident FP tumors vs turtles with evident tumors kept in varying UV light conditions at the rehabilitation facility at GLNC. As the animals in this facility are not routinely sampled for the presence of ChHV5, observation alone cannot determine if they are ChHV5 positive. Thus, for the purposes of this study, we categorized animals by the presence or absence of visible tumors (VT+/VT−) without presumption of viral infection [19]. If turtles were found to have internal nodules post-mortem, they were then placed in the VT+ category. Samples were also collected from presumed healthy turtles caught at the St. Lucie Power Plant by IRG; all power plant turtles used in this study were thus VT−. For ease of reference, the turtles used in this study were thus divided into three main groups: turtles at GLNC for rehabilitation with evident tumors (VT+), turtles at GLNC for rehabilitation without evident tumors (VT−), and healthy turtles caught by IRG, also without evident tumors.

### 2.1. GLNC Candidate Selection and Care

Juvenile green turtles appropriate for the study (see below) that were either with (VT+) or without (VT−) visible tumors were placed upon admittance to GLNC into one of four groups (VT− control (*n* = 5), VT− treatment (*n* = 6), VT+ control (*n* =5), or VT+ treatment (*n* = 7)), with control and treatment placement randomized within the VT+ and VT− groups, with treatment defined as exposure to higher levels of sunlight. All turtles brought to GLNC were assessed by the attending veterinarian and GLNC staff to determine inclusion in the study. To minimize variability due to secondary anomalies, turtles with severe secondary injuries (i.e., invasive boat strike, deep lacerations from constricting entanglement, etc.) were excluded. Turtles with minor secondary injuries (healed boat strike, superficial entanglement, etc.) were considered for inclusion. Turtles included in this study were juveniles ranging from >25 cm to <60 cm straight carapace length. 

All turtles were cared for by GLNC rehabilitation staff and received routine veterinary care. Animals were offered a consistent diet of up to 5% BW daily of the same variety of silversides, squid, shrimp, caplin, and green vegetation, as directed by the attending veterinarian. Every attempt to eliminate variation due to diet was made by providing the same food for all individuals. Standard medical interventions were made as needed, these included fluids, antibiotics, and vitamins. Routine care also included daily administration of a vitamin D gel capsule treatment (5000 IU), which likely altered vitamin D levels. The dose was standardized and used as a baseline for all turtles in the study, with UVB (sunlight) treatment potentially increasing vitamin D levels further. 

The depth of the water in the tanks varied depending on the individuals’ condition. Upon intake, most individuals were kept in tanks with a water depth of about 15 cm. As their condition improved, the water depth was raised to 40 to 60 cm depth depending on the size of the tank. The aquaria utilize a flow-through system with water pumped directly from the ocean offshore, which is filtered through both a sand and UV filter. The flow rate into the tanks averages ~600 L/h with a temperature ranging from 22.1 °C to 29.9 °C depending on the time of year (mean 26.9 °C). The water temperature of the tanks does not vary much from the mean due to the constant water flow through the system. 

### 2.2. IRG Candidate Selection and Sample Collection

Turtles (*n* = 12) captured at the St. Lucie Power Plant by IRG are individuals that were entrained into the inflow area. The turtles were collected by IRG and body measurements and a blood sample (~5 mL) were taken, prior to the turtle being tagged and released. All individuals included from that source were healthy VT− juveniles ranging from >25 cm to <60 cm straight carapace length. 

### 2.3. UV Light Treatment

UV light treatment was only given to turtles kept at GLNC. Turtles were exposed to varying amounts of UV light depending on whether the tank was in full sun (treatment groups) or was under cover and thus received only indirect or angled sunlight (control groups). The amount of light the tanks received daily depended on the sun exposure each day and varied with angle, cloud cover, and time of year. The UVB radiation was measured with a HOBO radiometer-photometer (Onset Computers, Bourne, MA, USA) at 30-min intervals at the water’s surface in all tanks to compare maximum potential UVB exposure. Although this measured the maximum amount of UVB exposure, the actual amount of UVB radiation each turtle received varied depending on behavior, as they would spend some time submerged in the water during the day. All turtles were monitored by rehabilitation staff for adverse reactions to the treatment; however, no such reactions occurred.

### 2.4. Blood Sample Collection

All samples from rehabilitation turtles were collected by GLNC staff under MTP #084. Blood samples were taken upon intake, prior to release and at 2-month intervals if the animal remained longer than 8–12 weeks. Prior to blood sampling, skin was disinfected 3 times with isopropyl alcohol. A sample of ~5 mL of blood was drawn from the dorsal cervical sinus with a 21 or 22 gauge needle into a vacutainer. All samples were significantly less than the safe maximal amount of blood that can be drawn from a healthy reptile, determined to be 4% of body weight converted to volume, or 3 mL/kg [42,43]. Blood was drawn regularly during the rehabilitation process for in house blood chemistry analyses and for cell blood counts (CBC) and differentials, which were determined by the University of Miami Health Systems Comparative Pathology Laboratory. For this study, ~2.5 mL of blood was placed in a lithium-heparin tube and spun down to separate the plasma. To measure vit D levels, ~1.5 mL of plasma was collected and stored at −80 °C until shipped to Animal Health Diagnostic Laboratory at Michigan State University where 25-hydroxyvitamin D3 concentrations were determined. 

### 2.5. Post-Surgical Monitoring for Regrowth 

VT+ animals at the facility were kept for varying amounts of time ranging from about 8 to 22 weeks prior to and after undergoing tumor removal surgery, dependent on overall animal condition and tumor severity. Turtles were monitored post-surgery for any tumor regrowth. The individuals were checked every two weeks and the number of tumors that regrew were recorded and photographed. 

### 2.6. Body Condition Index Calculations

Body condition index was determined for each individual upon intake to provide a standardized way to compare the variation seen among patients. Body condition index was calculated by dividing the weight (kg) of the individual by the straight carapace length (cm) cubed and multiplying it by 10,000 [44].

### 2.7. Statistical Analysis

Comparisons were made between control and treatment animals and between VT− (GLNC and/or IRG turtles) and VT+ groups. Data were tested for normality utilizing the Shapiro–Wilk test, and one-way analysis of variance (ANOVA) and *t*-tests were used to detect differences between groups. Data that were not normally distributed were analyzed via non-parametric tests: Kruskall–Wallace with Dunn’s test and Wilcoxon rank sum test, respectively. Correlations were also run to compare vitamin D levels with body condition index. Pearson correlations were run when data were normally distributed, and Spearman rank correlations were run when data were not normally distributed. 

## 3. Results

### 3.1. Hematology and Blood Chemistry Parameters 

**Figure 1:**
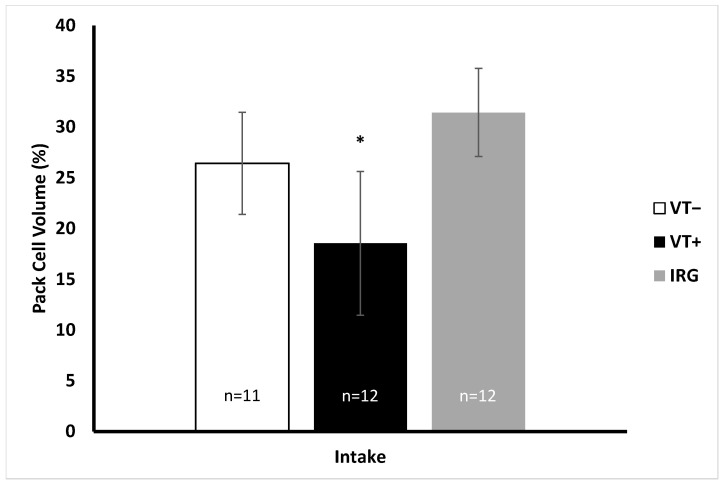
Intake packed cell volume (PCV) for turtles at the rehabilitation facility (VT+/VT−) and wild-caught (IRG) turtles. * VT+ turtle PCV values were significantly different from VT− and IRG turtles (*p* < 0.001). Error bars are one standard deviation from the mean.

Packed cell volume (PCV), white blood cell count (WBC), and leukocyte differentials were determined for all samples collected from turtles in rehabilitation. Only PCV was recorded for IRG turtles. PCV values at intake/capture were highest in IRG turtles (range 24.5–42.5%) and lowest in VT+ turtles (range 5–27%), with VT− turtles intermediate (20–39.5%) (Figure 1). Even though VT− turtles had intermediate values, there was a significant difference between VT+ and both IRG and VT− turtles (*p* < 0.005). WBC count ranged from 7600–19,800 cells in VT− turtles and 7300–49,000 cells in VT+ turtles. Median WBC count was higher in VT+ turtles than VT− turtles; however, the difference was not significant (*p* > 0.05). The population distribution of leukocytes was consistent between VT− turtles and VT+ turtles, and there was no significant difference between VT− and VT+ turtles (*p* > 0.05). 

Blood chemistry values were measured upon arrival for sea turtles brought in for rehabilitation at GLNC as part of standard intake procedures. Chemistry parameters, except for glucose, calcium, albumin, and cholesterol, were not significantly different between VT− and VT+ turtles (Table 1). Glucose, calcium, albumin, and cholesterol values were all significantly higher in VT− turtles than in VT+ turtles (*p* < 0.05). Correlations with body condition index showed individuals with better body condition had higher levels of calcium (*p* < 0.05); however, there was no significant correlation between body condition index and glucose, albumin, or cholesterol (*p* > 0.05). 

### 3.2. UV Light Exposure Conditions

**Figure 2:**
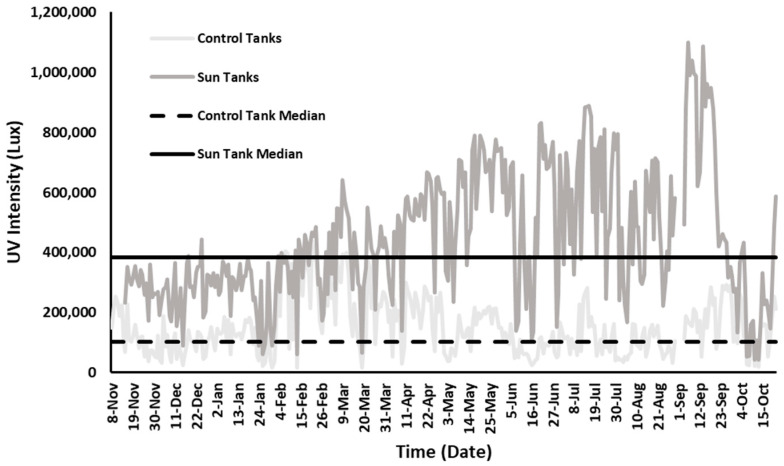
Daily fluctuations in UV intensity for control (low sun exposure) and treatment (high sun exposure) tanks over the course of a year. Median values for the control tanks and sun treatment tanks are significantly different (*p* < 0.001).

The potential for UV light exposure varies throughout the rehabilitation facility at GLNC. UV intensity varies diurnally as well as throughout the year (Figure 2). The daily median for tanks included in this study ranged from 54,649 Lux to 184,720 Lux in low UV light tanks (covered, controls) and from 356,438 Lux to 429,084 Lux in high UV light (sun, treatment) tanks. There was a significant difference in the daily median UV intensity between the control tanks and the sun tanks (*p* < 0.001) as well as seasonal variations. In the sun tanks, UV intensity was significantly greater in summer months compared to winter months (*p* < 0.01); however, in control tanks the opposite was true as UV intensity was significantly greater in winter months than in the summer (*p* < 0.01). The control tanks were located on the south side of the covered area in the facility, and as a result the lower angle of the sun in winter increased sun exposure compared to summer. Both the median and daily UV intensity in the fully exposed sun tanks, however, were always greater than the control tanks, even with the seasonal changes.

### 3.3. Vitamin D, Parathyroid Hormone, and Ionized Calcium

**Figure 3:**
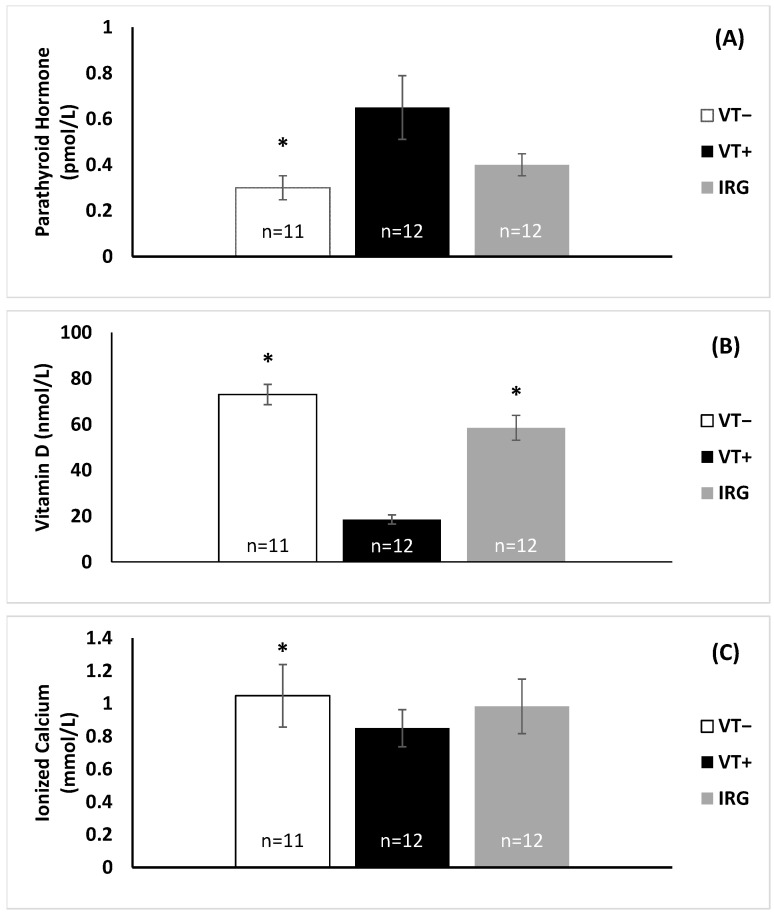
Parathyroid hormone (**A**), vitamin D (**B**), and ionized calcium (**C**) levels on intake for turtles taken into rehabilitation with (VT+) and without (VT−) papilloma tumors and in healthy wild-caught (IRG) individuals. * Indicates groups that were significantly different from the VT+ turtles (*p* < 0.05). Error bars are one standard error from the median for (**A**,**B**). Error bars were one standard deviation from the mean for (**C**).

Levels of parathyroid hormone (PTH), ionized calcium, and plasma 25-hydroxyvitamin D were similar to those reported in previous studies. PTH levels ranged from not detectable (ND)-1 pmol/L in VT− turtles, 0.3–1.4 pmol/L in VT+ turtles, and 0.3–2.8 pmol/L in IRG turtles (Figure 3A). For statistical purposes, samples below the minimum PTH detection threshold of 0.2 pmol/L were treated as midway between zero and the minimum detection limit (0.1 pmol/L). The median PTH level was significantly higher in VT+ animals compared to the VT− animals (*p* < 0.05), but there was no significant difference between IRG animals and VT+ or VT− animals. (*p* > 0.05). On the other hand, vitamin D levels were significantly lower in VT+ turtles compared to both groups of turtles without evident tumors (VT− and IRG) (Figure 3B, *p* < 0.001). There was no significant difference in vit D levels between VT− and IRG individuals (*p* > 0.05). Similarly, ionized calcium levels were significantly lower in VT+ turtles upon intake compared to VT− turtles (Figure 3C, *p* < 0.01); but there was no significant difference (*p* > 0.05) between IRG animals and VT− or VT+ turtles. 

**Figure 4:**
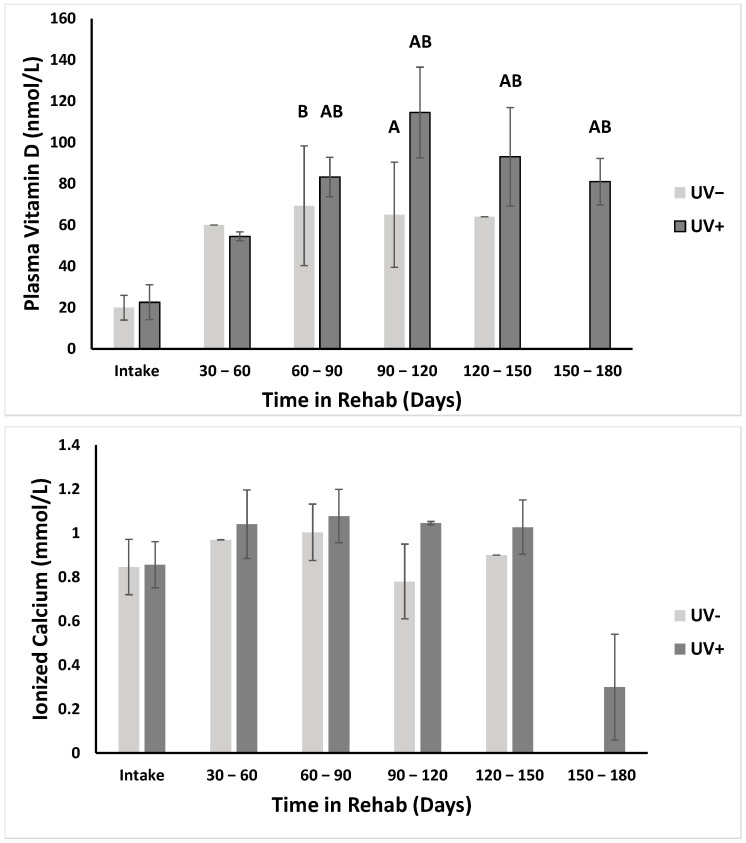
Change in plasma vitamin D (top) and ionized calcium (bottom) levels over time in turtles taken into rehabilitation with papilloma tumors (VT+). **A** = significant difference from UV− intake values. **B** = significant difference from UV+ intake values. *p* < 0.05.

As the objective of this study was to determine if UV light influenced vitamin D levels and light levels varied significantly depending on which tanks the turtles were kept in, we examined changes in vitamin D over time. VT+ turtles admitted to GLNC for treatment had both lower vitamin D levels at intake and generally stayed longer in rehabilitation than VT− turtles, as their injuries/disease required longer intensive care, so the greatest changes were seen in these animals (Table 2). VT+ turtles kept in high UV light conditions had greater increases in vitamin D levels over time compared to the VT+ turtles kept in the low UV light conditions (Figure 4A). There was a significant increase in vit D for VT+ turtles exposed to high UV light when their recovery lasted longer than 60 days (*p* < 0.05). VT+ turtles exposed to low UV light conditions experienced a slight increase in vitamin D, but it was not significant (*p* > 0.05). A similar trend was seen in VT− turtles. VT− turtles kept in high UV light conditions had greater vitamin D levels over time compared to those kept in low UV light conditions, though the change over time was also not significant (*p* > 0.05). We also investigated how parathyroid hormone and ionized calcium varied over time for individuals undergoing rehabilitation. Parathyroid hormone showed a reduction over time in VT+ turtles and no variation in VT−; however, none of the values varied significantly for VT+ or VT− turtles exposed to low or high UV light (*p* > 0.05). Ionized calcium levels increased slightly in VT+ turtles exposed to low and high UV light, but it did not vary in the VT− turtles. The variation over time was significant for VT+ turtles exposed to both low and high UV light (Figure 4B, *p* < 0.05); however, when running a post hoc test to determine which 30-day periods were significantly different, there was no significant difference among any of the groups (*p* > 0.05). There was also no significant difference over time for ionized calcium levels in VT− turtles exposed to low or high UV light (*p* > 0.05). Since each animal’s condition varied greatly upon intake, we calculated body condition index (BCI) for all individuals to determine if there was a correlation between BCI and vitamin D levels upon presentation; however, there was no significant correlation (*p* > 0.05). 

### 3.4. Observations on Regrowth and Survival

**Figure 5:**
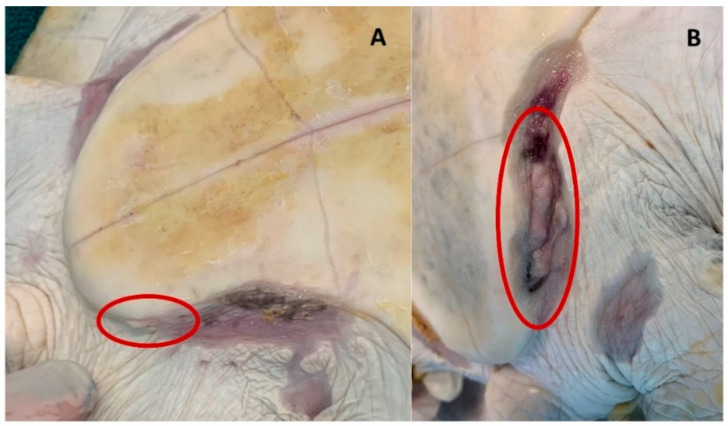
Representative images of regrowth of the left inguinal region of the plastron in two different individuals. (**A**) Represents regrowth of a VT+ UV+ individual 7 weeks post-surgery. (**B**) Represents regrowth of a VT+ UV− individual, 10 weeks post-surgery.

Since animals both with and without visible tumors entered the rehabilitation facility and were utilized in this study, we thought it would be of interest to determine if there was any correlation between sun exposure, vitamin D, and tumor regrowth, thus, the turtles which had tumor removal surgery were monitored for any regrowth. The turtles in this study had tumors located over all the body, with the most common areas affected being along the soft tissue of the flippers and on the eyes. Of the 7 turtles that underwent surgery, 4 turtles experienced regrowth. All of the turtles that experienced regrowth had a Balazs tumor score of 3 on intake. The 3 turtles that did not experience regrowth had tumor scores of 1, 2, and 3. The number of regrowth areas ranged from 5 to 13, and the most common area for regrowth seen in these individuals was along the soft tissues, especially in the inguinal area. Regrowth seen in these turtles occurred within a month post-surgery. Turtles kept in high UV light conditions (treatment tanks) appeared to experience less regrowth (both fewer and smaller regrowth areas) than those kept in low UV light (control) conditions (Figure 5), though as the sample size is small, we consider this a preliminary observation only and in need of further study.

Similarly, the outcomes for the individuals brought into rehabilitation varied: VT− individuals (11/11) were all released. For VT+ individuals, the majority were euthanized or died and only 5 of 12 were released. The survival of VT+ turtles appears to be greater when exposed to higher UV light (4/5 released) compared to those exposed to low UV light conditions (1/7 released); however, the sample size again is too small for statistical analysis and further research is needed. 

## 4. Discussion

This study focused on the effect of UV light on vitamin D levels in green sea turtles with FP, a disease that affects up to 60% of individuals in some sub-populations [45,46]. When brought into rehabilitation facilities, the animals are treated via the surgical removal of external tumors. Although the tumors can be removed, the prognosis for turtles with this disease is poor. A way to better the outcome for VT+ turtles could improve rates of survival and release. In this study, we examined several health parameters in VT− versus VT+ turtles, as well as comparing values to wild-caught turtles without tumors. While higher UV light exposure had no effect on PTH or ionized calcium levels, animals in sun tanks showed improved vitamin D levels over time, which could have a variety of health benefits and may have influenced the regrowth of fibropapilloma tumors.

### 4.1. Hematology and Blood Chemistry Parameters

It was important to determine if there were any variations in baseline health parameters for the turtles in this study, because turtles brought into GLNC come from all along the east coast of Florida. They are exposed to different environmental conditions and may be suffering from a variety of health issues. The turtles that come into the St. Lucie power plant (IRG group) are likewise exposed to different environmental conditions, but previous life history was not something we could control for in this study. We did compare body condition index (BCI) between VT+ and VT− animals, and while VT+ turtles had a slightly lower BCI upon intake, the difference was not significant. Packed cell volume (PCV or hematocrit) varied between rehabilitation turtles upon intake and IRG turtles (Figure 1). By definition, all individuals admitted into GLNC were dealing with some type of disease or injury which likely resulted in reduced PCVs. Bloodgood et al. similarly found that turtles brought into rehabilitation facilities had significantly lower PCVs compared to wild individuals [41]. In this study, significant differences in PCV occurred between VT+ and both VT− and IRG turtles, where VT+ turtles had the lowest PCV value, VT− turtles had intermediate PCV values, and IRG turtles had the greatest. This is not surprising since the VT− population was selected to be those animals without severe injury or illness, thus, the minor health problems (e.g., recently swallowed fish hook) would have minimal impacts on this measure of health. The trends observed here are similar to results reported in other studies, where VT+ turtles have lower PCV values when compared to healthy individuals [17,47,48,49]. In Hawaii, turtles with the most severe Balazs tumor scores also had the lowest PCV values, especially in comparison to VT− individuals [48]. Free-living turtles from the Indian River Lagoon have also been shown to have an inverse relationship between PCV values and tumor score, with the lowest PCV values seen in turtles with a tumor score of 3 [49]. These individuals with FP, including the ones in this study, are in poor condition and often anemic, which results in lower PCV values [17,47]. Other hematological parameters also appear to be influenced by this disease. 

White blood cell counts in several studies have also been shown to vary between VT− and VT+ individuals. Cray et al. found that turtles with FP had higher WBC counts compared to turtles without FP [16]. Aguirre et al., on the other hand, saw higher WBC counts in VT− turtles compared to VT+ turtles [17]. Higher WBC counts typically suggest the body is fighting off some type of infection [49]. Differences seen in these studies could potentially be due to differences in environmental conditions, as well as varying underlying injuries or disease. Although in this study there was no significant difference between VT− and VT+ WBC count values overall, VT+ turtles had a greater number of heterophils as a percentage of the total cell population. Heterophils are one of the main phagocytotic cells that respond to inflammation and infection [50]. The differences in heterophil counts between VT+ and VT− turtles was not significant, but our results follow the same trends as in previous studies. Cray et al. found that captive VT− turtles’ leukocyte populations were significantly lower than in VT+ turtles [16], while Sposato et al. reported that heterophils comprised the largest fraction of the leukocyte population in green sea turtles from both a polluted and a more pristine Florida environment [19]. Increased heterophil counts indicate an inflammatory response or, in the case of VT+ turtles, a response to tumors or agents related to the tumors [51]. 

We found that some blood chemistry parameters also varied between VT+ and VT− turtles, though the majority showed no significant differences. Calcium, albumin, and cholesterol were significantly lower in VT+ turtles compared to VT− turtles (Table 1), as was also reported by Aguirre et al. and Aguirre and Balazs [17,52]. Lower glucose levels in VT+ turtles were seen in those studies as well; however, unlike the current study, the difference was not significant. Low values for these chemistry parameters are typically associated with severe debilitation, which is common for turtles with FP; in this study, only calcium was significantly correlated with body condition index. In a previous study by Bloodgood et al. [41], ionized calcium levels were similarly significantly lower in green turtles brought into a rehabilitation facility (in Georgia, USA) than those free-ranging and captured, as ours were, at the St. Lucie Power Plant in Florida (0.85 ± 0.18 mmol/L vs. 1.01 ± 0.23 mmol/L), while in Hawaii plasma calcium in green turtles was in the range 0.27–3.02 mmol/L [52] with a mean of 2.3 ± 0.42 mmol/L. Calcium levels may also have been influenced by critical hormone levels that play a role in calcium homeostasis.

### 4.2. Baseline Vitamin D Levels

Vitamin D plays a well-known role in calcium homeostasis, but is also known to influence the immune system, thus, we examined how levels of plasma vitamin D were influenced by UV light exposure. Vitamin D can be obtained through the skin when it is exposed to UV light or through the consumption of foods that contain vitamin D_2_ or D_3_ [53]. Studies of both mammals and reptiles show greater vitamin D levels when animals are exposed to sunlight. Dairy cows exposed to natural sunlight or artificial UVB light for 73 days had increased plasma vitamin D levels [54], for example, while red eared slider turtles provided UV supplementation had significantly higher plasma vitamin D levels after 30 days compared to individuals kept only in ambient light [36]. Corn snakes also showed higher plasma vitamin D levels when exposed to supplemental UV light [35], as did agamid and iguanid lizards exposed to natural sunlight vs. those exposed only to artificial lights indoors [37]. Several studies in lizards suggest that these reptiles can tightly regulate sun exposure to raise vit D levels, with vit D increasing significantly upon exposure to UVB rays, and those animals with low dietary vit D sought out stronger UV light [55,56]. As with other reptiles, vitamin D is a necessary nutrient for green sea turtles [57]; however, few studies have looked at baseline levels in this species or the effects of UV exposure on vitamin D.

The baseline values for vitamin D, parathyroid hormone, and ionized calcium levels have only been determined in green sea turtles in a few studies, and thus, the reference range of vitamin D levels and other blood chemistry values in green sea turtles is not truly known. Few studies have met the standard large sample size required for true reference ranges, though Flint et al. [58] found plasma ionized calcium to range from 0.2 to 2.2 mmol/L in wild green turtles in Australia, while Aguirre and Balazs [52] report a reference range of 0.27–3.02 mmol/L; our plasma calcium values were within this range. Most studies have looked at vit D values in captive individuals, where they are typically lower due to limited exposure to UVB irradiation. The few studies that have compared captive to free-ranging show free-ranging individuals to have an average vitamin D level somewhere between 36 and 46.55 nmol/L depending on the study [40,41]. These studies tend to show a large range in the values, sometimes ranging from 16.1 to 72.1 nmol/L [40,41]. Our values for vit D in VT− and IRG turtles were in the range of 44–99 nmol/L and 36–108 nmol/L, respectively, though VT+ turtles had significantly lower levels in the range of 12–33 nmol/L. Purgley et al. found that adult captive green turtles exposed to sunlight had vitamin D levels ranging from 61 to 69 nmol/L, while captive adults kept indoors had levels ranging from 5 to 53 nmol/L [49]. Purgley et al. also showed that the longer the animals were kept indoors without exposure to UV the lower the plasma vitamin D levels [39]. Two other studies have compared vitamin D levels, parathyroid hormone, and ionized calcium levels in rehabilitated versus wild-caught juvenile green sea turtles [40,41]. As with this study, Bloodgood et al. reported that turtles had lower vitamin D and ionized calcium levels upon admittance to rehabilitation than free-ranging, presumably healthy individuals, though unlike in our study, PTH levels were also low [41]. In the Stringer et al. study, turtles were in rehabilitation for 75–329 days with limited exposure to UVB light [44]. Despite an oral vitamin D_3_ supplement provided 3 × per week, these turtles had lower median vitamin D and ionized calcium values than wild-caught individuals. Although the difference in vitamin D levels between rehabilitated and wild-caught turtles was not significant, the reported trend mirrors our results, where less exposure to natural UV light resulted in lower vitamin D levels. PTH levels, however, were greater in the turtles brought into the rehabilitation facility compared to wild-caught individuals [44], as we saw with VT+ turtles compared to VT− animals either brought into GLNC or free-ranging (Figure 3A). Parathyroid hormone helps to regulate the amount of calcium in the blood such that when ionized calcium levels are low PTH is released, while vit D increases calcium absorption in the body [59]. The elevated PTH levels in our VT+ turtles coupled to the low levels of vitamin D and ionized calcium suggests that these individuals are vitamin D deficient, which in turn reduces calcium uptake and stimulates the release of PTH. Low vit D and ionized calcium could potentially be contributing to the poor health of individuals with FP. Adding UV light supplementation to the rehabilitation of VT+ and VT− turtles could thus positively influence vitamin D levels and improve outcomes. 

This is further suggested when comparing VT− turtles on intake to presumed healthy free-ranging turtles, where there were no significant differences in vitamin D levels, PTH, or ionized calcium levels between VT− and IRG individuals (Figure 3A–C). Ionized calcium levels were comparable to the baseline levels determined by Stringer et al. and Bloodgood et al. [44,45]; however, vitamin D levels were higher and PTH levels lower than in those studies. This may be due to the fact that most of our VT− turtles were hooked by fisherman at a nearby pier and were relatively healthy, whereas in other studies animals brought into rehabilitation are often quite ill or severely injured. Environmental conditions could also cause differences between studies. The turtles used by Stringer et al. were all from North Carolina, for example, where UV intensity is less than in southern Florida [43]. It has been shown in other vertebrates that latitude has an effect on plasma vitamin D levels, where animals at higher latitudes tend to have lower levels [60,61]. To our knowledge, the rehabilitated turtles included in the previous studies were not VT+ turtles, so determining baseline values in these turtles is needed.

Vitamin D levels increased over time in both VT+ and VT− turtles, with the most significant increases seen when the turtles were exposed to higher levels of UV light (Figure 4A) and for longer times. While the turtles exposed to lower UV light had an increase in plasma vitamin D levels, which is likely due in a large part to the oral vitamin D supplementation, the increase was not significant. The turtles exposed to higher UV light in addition to the oral supplement had much greater increases in plasma vitamin D levels. A study in bearded dragons similarly found that individuals given both an oral supplement and exposure to high UV light had much higher plasma vitamin D levels than those provided oral supplementation alone [38]. In this study, the changes in vitamin D were only significant in the VT+ turtles; while the VT− turtles showed the same trend, the changes were smaller and not significant. The lesser change in vit D in the VT− turtles is likely due to their short stay in rehabilitation, as well as higher vitamin D levels upon intake compared to the VT+ turtles. As vitamin D in green sea turtles does appear to increase with exposure to greater UV light, understanding the role it plays in their overall health in general could be important. 

As with vitamin D, ionized calcium levels increased slightly over time in VT+ turtles. The increase was significant when using a nonparametric Kruskall–Wallace test; however, when using the Dunn’s post-hoc test to compare groups, there was no significant difference between individual groups. The conflicting statistical results are likely due to the high variance seen in the data set. Since the Dunn’s post-hoc test is a stronger test, it is more accurate to conclude there was no significant change over time, though the trend seen in the data corresponds with the increasing vitamin D levels, which is to be expected. Vitamin D helps with the uptake of calcium in the intestines [53], so as vitamin D in the body increases, calcium levels should increase as well, assuming dietary sufficiency. Parathyroid hormone levels also follow the expected physiological trend. Although the change over time was not significant, PTH levels did decrease slightly in VT+ turtles exposed to both high and low UV light. The levels for VT− turtles remained relatively consistent over time in both UV groups. A decrease in PTH would be expected as the hormone is released when ionized calcium levels are low [59]. Since we saw an increase in ionized calcium and vitamin D, PTH could be expected to decrease as part of homeostasis. 

### 4.3. Regrowth, Viral Load, and Survival 

Regrowth of FP tumors is a common occurrence for turtles that have tumor removal surgery. One study showed that 50% of individuals that underwent tumor removal surgery experienced regrowth [21]. Of the turtles that underwent surgery in this study, 57% of individuals (4/7) experienced regrowth within a month post-surgery. All of the turtles that experienced regrowth had a Balazs tumor score of 3 upon intake. It is possible that tumor score could affect regrowth; however, the sample size is small, and a previous study found no significant relationship between tumor score and regrowth occurrence [21]. We did note a difference in regrowth depending on the exposure to UV light the turtles received. Those kept in the sun tanks experienced less regrowth compared to those exposed to low UV light conditions, as similarly occurred in a study in mice with mammary tumors [28]. Again, however, the sample size is too small in this study to determine any statistically significant relationship. In addition, every veterinarian uses a different technique to remove tumors. The most common form of tumor removal surgery is using a CO_2_ laser, however, some individuals use a cauterizer or scalpel blade to remove the tumors, resulting in different amounts of tissue damage. The amount of tissue that is removed around the tumor also depends on the veterinarian and can influence the amount of regrowth as well. In this study, a single veterinarian conducted all of the surgeries, though a combination of scalpel, cauterizer, and CO_2_ laser were used to remove the tumors. The method selected varied on individual turtles as it was based on the location and vascularization of the tumor, as well as the tolerance of the animal while under anesthesia. The use of a blade alone for tumor removal has a greater chance for regrowth, if tumor cells are not completely removed. Adding cautery or a CO_2_ laser can help kill tumor cells and prevent regrowth. All of these factors could have influenced the regrowth of tumors.

Besides tumor regrowth, the survival of green sea turtles with FP is significantly reduced compared to VT− turtles brought into rehabilitation facilities. One previous study showed that 75% of turtles admitted into rehabilitation facilities for FP either died or were euthanized [21]. Another study reported that 77.3% of turtles treated for FP died or were euthanized [22]. A similarly high number of VT+ turtles (64%) died or were euthanized in this study. Veterinarians at each rehabilitation facility also use their own judgement when it comes to euthanasia, however, and their criteria could differ and thus affect survival rates. Our preliminary results suggest that in those animals in a good enough condition to be candidates for surgery, UV light may have an effect on the survivorship of turtles with FP. Of those kept in low UV light conditions, 88% of VT+ turtles died or were euthanized, whereas only 33% of VT+ turtles kept in high UV light conditions died or were euthanized. However, with such a small number of animals the results could simply be random. Although the turtles were randomly assigned to tanks, three of the VT+ turtles placed in the low UV light tanks were later determined to have internal tumors and were euthanized, thus increasing the number of euthanized individuals in the VT+/low UV light group. Some of the turtles also came in with minor secondary conditions; other health parameters and the standard treatment of antibiotics and vitamins could have thus contributed to their chances of survival or lack thereof. 

Although the results of this study suggest that VT+ turtles housed in higher UV light conditions had better outcomes, a previous study by Duffy et al. suggests that UV light may increase FP [62]. That study compared gene expression in the FP tumors to various cancers in humans and found that these tumors are most closely related to basal cell carcinoma [62]. It was also observed that there was a significant positive correlation between UV light intensity and FP prevalence [62]. Although this correlation is present, it is not necessarily the UV light alone that is affecting FP. Most of the turtles that are found with FP are juvenile green sea turtles, which like to spend most of that stage of their life in nearshore areas [63]. These nearshore regions are exposed to a number of anthropogenic factors, especially pollution. Polluted environments have been shown to suppress the immune system, and residence in nearshore environments can expose these turtles to a greater level of pollution at this life stage [19]. This in turn could be increasing the number of VT+ cases. Water temperatures in these high UV nearshore areas are often higher than offshore [64] and also may impact the prevalence of this disease as well as be a potential stressor for these individuals. This study provides preliminary evidence that there is a benefit to UV radiation, but further research with larger sample sizes is needed to determine the true effect on recovery.

### 4.4. Limitations of the Study

As with other somewhat opportunistic studies on wildlife, there are certain limitations to the conclusions of this study. While the sample size was sufficient to see statistically significant differences in blood chemistry parameters, as well as a significant change in vit D with increased sun exposure, too few animals with FP were admitted into the rehabilitation facility during the study period to make any definitive link between vitamin D levels and recovery from FP surgery. Thus, these observations in particular are presented as preliminary only and suggested as an area for further study. The statistically significant correlation between the presence of tumors upon admittance and vit D levels does suggest a link between health and FP, as has been suggested in other animal studies; however, the physiology of vit D in reptiles has been poorly studied beyond its relation to bone health, egg mineralization, and embryonic survival [37,65]. In other animal studies, vit D has been linked to better immune function, including the inhibition of viruses [23] and enhanced phagocytosis [33] and antimicrobial activity [32,34]. However, while the blood chemistry, PCV, and BCI suggest that VT− turtles brought in for rehabilitation were physically comparable to the wild-caught turtles and WBC counts were not different between VT+ and VT− animals, the sample size was too small for any measure of immune function that would have provided a more direct indication of health either upon intake or after treatment. As immune function data can be quite variable, a large number of animals would be required to reach statistical significance; many immune function studies in sea turtles have sample sizes of approximately 45 to 85 animals [16,19,66,67]. It would certainly be of interest to perform a larger study with a more controlled exposure to UV light, perhaps by dry-docking turtles, to look at the effects of sun exposure on immune parameters such as the strength of the phagocytic response or lymphocyte proliferation. 

## 5. Conclusions

Green sea turtles are an endangered species and are subject to a number of factors that hinder their survival. Fibropapillomatosis is one disease that affects these turtles worldwide. Although the disease can be treated with surgery, the outlook for turtles with this disease is poor and often results in euthanasia. When turtles are stable enough to undergo surgery, the majority experience regrowth of the tumors. Individuals with FP demonstrate reduced vitamin D levels and variations in blood chemistry parameters. Our data suggest that one potential method to better the outcome of turtles with this disease is increased exposure to UV light during rehabilitation. Although a cure for this disease has yet to be discovered, exposure to high UV light increases vit D in animal models and increased plasma vit D levels are in turn correlated with lower rates of disease; this potential to boost health could thus contribute to improved recovery. It would be of interest to determine if there are direct links between vitamin D levels and immune function in future studies.

## Figures and Tables

**Table 1:** Intake blood chemistry values for turtles taken into the GLNC rehabilitation facility. Glucose, calcium, albumin, and cholesterol values in VT− turtles were significantly different than VT+ values (*p* < 0.05). No other blood chemistry values were significantly different (*p* > 0.05). * Medians were used for these chemistries due to non-normalized data.

Chemistry Parameter	VT− Mean/ Median (*n* = 11)	VT− Range	VT+ Mean/ Median (*n* = 12)	VT+ Range	Significance
Glucose	130	102–183	78.6	16–156	*p* < 0.01
BUN	49	2–105	60.9	16–130	n.s.
Uric Acid *	0.9	0.5–4.5	1.5	0.4–3.3	n.s.
Phosphorus	7.1	4.7–11.8	7.4	2.7–10.5	n.s.
Calcium *	7.5	6.1–11.1	5.5	4.1–6.3	*p* < 0.001
Sodium	155.7	148–161	153.7	149–159	n.s.
Potassium	4.2	2.9–4.7	4.3	3.4–5.2	n.s.
Na:K Ratio *	35.5	33–53	36	30–46	n.s.
Chloride	130.6	122–141	125.6	116–135	n.s.
Total Protein	4	1.9–5	3.4	2.3–4.6	n.s.
Albumin	1.4	0.8–2	1.1	0.5–1.5	*p* < 0.05
Globulin	2.6	1.1–3.6	2.2	1.5–3.1	n.s.
Albumin:Globulin Ratio	0.55	0.4–0.7	0.53	0.3–0.8	n.s.
ALT *	14	10–102	11	2–212	n.s.
ALP *	23	10–52	17.5	10–52	n.s.
GGT *	0	0–3	0	0–1	n.s.
Bilirubin *	0.2	0.1–0.4	0.1	0.1–0.5	n.s.
Cholesterol *	85.5	55–152	9	6–119	*p* < 0.001
Amylase	396	178–696	333	77–723	n.s.
Lipase *	13.5	10–33	21	10–88	n.s.
Osmolality	322.5	292–339	312.3	300–328	n.s.

**Table 2:** Number of individuals sampled in each treatment group at various time points while residing at GLNC. Time points were grouped into 30-day periods. Some individuals were sampled multiple times depending on the duration they remained at GLNC. Some VT+ individuals died or were euthanized shortly after intake and so were sampled only once.

Group	Intake	<30 Days in Rehab	30–60 Days in Rehab	60–90 Days in Rehab	90–120 Days in Rehab	120–150 Days in Rehab	150–180 Days in Rehab
VT−UV−	5	2	3	1	0	0	0
VT−UV+	6	1	4	1	0	0	0
VT+UV−	7	0	1	3	2	1	0
VT+UV+	5	0	2	4	2	3	2

## Data Availability

Data will be available upon on request from the corresponding author. Data are not publicly available as the data may be used as components of continued research at GLNC and FAU.

## References

[1] Smith G.M., Coates C.W. (1938). Fibro-epithelial growths of the skin in large marine turtles, *Chelonia mydas* (Linnaeus). Zoologica.

[2] Harshbarger J.C., Balazs G.H., Pooley S.G. (1991). Sea turtle fibropapilloma cases in the registry of tumors in lower animals. Research Plan for Marine Turtle Fibropapilloma: Results of a December 1990 Workshop.

[3] Barragan A.R., Sarti M.L. (1994). A possible case of fibropapilloma in Kemp’s Ridley turtle (*Lepidochelys kempii*). Mar. Turt. Newsl..

[4] D’Amato A.F., Moraes-Neto M. (2000). First documentation of fibropapillomas verified by histopathology in *Eretmochelys imbricata*. Mar. Turt. Newsl..

[5] Aguirre A.A., Spraker T.R., Chaves A., Toit L., Eure W., Balazs G.H. (1999). Pathology of fibropapillomatosis in Olive Ridley turtles *Lepidochelys olivacea* nesting in Costa Rica. J. Aquat. Anim. Health.

[6] Limpus C.J., Couper P.J., Couper K.L.D. (1993). Crab Island revisited: Reassessment of the world’s largest Flatback turtle rookery after twelve years. Mem. Queensl. Mus..

[7] Jones K., Ariel E., Burgess G., Read M. (2016). A review of fibropapillomatosis in Green turtles (*Chelonia mydas*). Vet. J..

[8] Jones A.G. (2004). Sea Turtles: Old Viruses and New Tricks. Curr. Biol..

[9] Adams M.J., Carstens E.B. (2012). Ratification vote on taxonomic proposals to the International Committee on Taxonomy of Viruses. Arch. Virol..

[10] Page-Karjian A., Norton T.M., Ritchie B., Brown C., Mancia C., Jackwood M., Gottdenker N.L. (2015). Quantifying chelonid herpesvirus 5 in symptomatic and asymptomatic rehabilitating green sea turtles. Endang. Species Res..

[11] Milton S.L., Lutz P.L., Lutz P.L., Musick J.A., Wyneken J. (2003). Physiological and genetic responses to environmental stress. The Biology of Sea Turtles.

[12] Aguirre A.A., Lutz P.L. (2004). Marine turtles as sentinels of ecosystem health: Is fibropapillomatosis an indicator?. EcoHealth.

[13] Foley A.M., Schroeder B.A., Redlow A.E., Fick-Child K.J., Teas W.G. (2005). Fibropapillomatosis in stranded green turtles (*Chelonia mydas*) from the eastern United States (1980-98): Trends and associations with environmental factors. J. Wildl. Dis..

[14] Dos Santos R.G., Martins A.S., Torezani E., Baptistotte C., Nobrega F.J., Horta P.A., Work T.M., Balazs G.H. (2010). Relationship between fibropapillomatosis and environmental quality: A case study with *Chelonia mydas* off Brazil. Dis. Aquat. Organ..

[15] Lutz P.L., Cray C., Sposato P.L. (2001). Studies of the Association between Immunosuppression and Fibropapillomatosis within Three Habitats of Chelonia mydas.

[16] Cray C., Varella R., Bossart G.D., Lutz P.L. (2001). Altered in vitro immune responses in green turtles (*Chelonia mydas*) with fibropapillomatosis. J. Zoo Wildl. Med..

[17] Aguirre A.A., Balazs G.H., Spraker T.R., Gross T.S. (1995). Adrenal and hematological responses to stress in juvenile green turtles (*Chelonia mydas*) with and without fibropapillomas. Physiol. Zool..

[18] Work T.M., Rameyer R.A., Balazs G.H., Cray C., Chang S.P. (2001). Immune status of free-ranging green turtles with fibropapillomatosis from Hawaii. J. Wildl. Dis..

[19] Sposato P., Keating P., Lutz P.L., Milton S.L. (2021). Evaluation of immune function in two populations of green sea turtles (*Chelonia mydas*) in a degraded versus a nondegraded habitat. J. Wildl. Dis..

[20] Page-Karjian A., Norton T.M., Krimer P., Groner M., Nelson S.E., Gottdenker N.L. (2014). Factors influencing survivorship of rehabilitating green sea turtles (*Chelonia mydas*) with fibropapillomatosis. J. Zoo Wildl. Med..

[21] Page-Karjian A., Perrault J.R., Zirkelbach B., Pescatore J., Riley R., Stadler M., Zachariah T.T., Marks W., Norton T.M. (2019). Tumor re-growth, case outcome, and tumor scoring systems in rehabilitated green turtles with fibropapillomatosis. Dis. Aquat. Organ..

[22] Stacy B.A., Foley A.M., Work T.M., Lauritsen A.M., Schroeder B.A., Hargrove S.K., Keene J.L. (2018). Report of the Technical Expert Workshop: Developing Recommendations for Field Response, Captive Management, and Rehabilitation of Sea Turtles with Fibropapillomatosis.

[23] Chao C.T., Chiang C.K., Huang J.W., Hung K.Y. (2015). Vitamin D is closely linked to the clinical courses of herpes zoster: From pathogenesis to complications. Med. Hypotheses.

[24] Beard J.A., Bearden A., Striker R. (2011). Vitamin D and the anti-viral state. J. Clin. Virol..

[25] Prietl B., Treiber G., Pieber T.R., Amrein K. (2013). Vitamin D and Immune Function. Nutrients.

[26] Barrett K.E., Brooks H.L., Boitano S., Barman S.M. (2010). Ganong’s Review of Medical Physiology.

[27] Trochoutsou A.I., Kloukina V., Samitas K., Xanthou G. (2015). Vitamin-D in the immune system: Genomic and non-genomic actions. Mini Rev. Med. Chem..

[28] Valrance M.E., Brunt A.H., Welsh J. (2007). Vitamin D receptor-dependant inhibition of mammary tumor growth by EB1089 and ultraviolet radiation in vivo. Endocrinology.

[29] Watkins R.R., Lemonovich T.L., Salata R.A. (2015). An update on the association of vitamin D deficiency with common infectious diseases. Can. J. Physiol. Pharmacol..

[30] Hewison M. (2012). Vitamin D and immune function: An overview. Proc. Nutr. Soc..

[31] Charoenngam N., Holick M.F. (2020). Immunologic effects of vitamin on human health and disease. Nutrients.

[32] De Smet K., Contreras R. (2005). Human antimicrobial peptides: Defensins, cathelicidins and histatins. Biotechnol. Lett..

[33] Dioguardi M., Guardiola F.A., Vazzana M., Cuesta A., Esteban M.A., Cammarata M. (2017). Vitamin D_3_ affects innate immune status of European sea bass (*Dicentrarchus labrax* L.). Fish Physiol. Biochem..

[34] Estévez R.A., Mostazo M., Rodriguez E., Espinoza J.C., Kuznar J., Jónsson Z.O., Guðmundsson G.H., Maier V.H. (2018). Inducers of salmon innate immunity: An in vitro and in vivo approach. Fish Shellfish Immunol..

[35] Acierno M.J., Mitchell M.A., Zachariah T.T., Roundtree M.K., Kirchgessner M.S., Sanchez-Migallon Guzman D. (2008). Effects of ultraviolet radiation on plasma 25-hydroxyvitamin D3 concentrations in corn snakes (*Elaphe guttata*). Am. J. Vet. Res..

[36] Acierno M.J., Mitchell M.A., Roundtree M.K., Zachariah T.T. (2007). Effects of ultraviolet radiation of 25-hydroxyvitamin D_3_ synthesis in red-eared slider turtles (*Trachemys scripta elegans*). Am. J. Vet. Res..

[37] Laing C.J., Trube A., Shea G.M., Fraser D.R. (2001). The requirement for natural sunlight to prevent vitamin D deficiency in iguanian lizards. J. Zoo Wildl. Med..

[38] Oonincx D.G.A.B., Stevens Y., Van den Borne J.J.G.C., Van Leeuwen J.P.T.M., Hendriks W.H. (2010). Effects of vitamin D_3_ supplementation and UVb exposure on the growth and plasma concentration of vitamin D_3_ metabolites in juvenile bearded dragons (*Pogona vitticeps*). Comp. Biochem. Physiol. B.

[39] Purgley H., Jewell J., Deacon J.E., Winokur R.M., Tripoli V.M. (2009). Vitamin D3 in Captive Green Sea turtles (*Chelonia mydas*). Chelonian Conserv. Biol..

[40] Stringer E.M., Harms C.A., Beasley J.F., Anderson E.T. (2010). Comparison of ionized calcium, parathyroid hormone, and 25-hydroxyvitamin D in rehabilitating and healthy wild green sea turtles (*Chelonia mydas*). J. Herpetol. Med. Surg..

[41] Bloodgood J.C.G., Norton T.M., Hoopes L.A., Stacy N.I., Hernandez S.M. (2019). Comparison of hematological, plasma biochemical, and nutritional analytes of rehabilitating and apparently healthy free-ranging Atlantic green turtles (*Chelonia mydas*). J. Zoo Wildl. Med..

[42] Lillywhite H.B., Smits A.W. (1984). Lability of blood volume in snakes and its relation to activity and hypertension. J. Exp. Biol..

[43] Smits A.W., Kozubowski M.M. (1985). Partitioning of body fluids and cardiovascular responses to circulatory hypovolaemia in the turtle, *Pseudemys scripta elegans*. J. Exp. Biol..

[44] Body Condition Scoring the Sea Turtle. https://lafeber.com/vet/body-condition-scoring-the-sea-turtle/.

[45] Herbst L.H. (1994). Fibropapillomatosis of marine turtles. Annu. Rev. Fish Dis..

[46] Hirama S., Ehrhart L.M. (2007). Description, prevalence and severity of green turtle fibropapillomatosis in three developmental habitats on the east coast of Florida. Fla. Sci..

[47] Zwarg T., Rossi S., Sanches T.C., Cesar M.O., Werneck M.R., Matushima E.R. (2014). Hematological and histopathological evaluation of wildlife green turtles (*Chelonia mydas*) with and without fibropapilloma from the north coast of São Paulo State, Brazil. Pesq. Vet. Bras..

[48] Work T.M., Balazs G.H. (1999). Relating tumor score to hematology in green turtles with fibropapillomatosis in Hawaii. J. Wildl. Dis..

[49] Hirama S., Ehrhart L.M., Rea L.D., Kiltie R.A. (2014). Relating fibropapilloma tumor severity to blood parameters in green turtles *Chelonia mydas*. Dis. Aquat. Org..

[50] Owen J.A., Punt J., Strandford S.A., Jones P.P. (2013). Kuby Immunology.

[51] Varela R.A. (1997). The immunology of green turtle fibropapillomatosis. Master’s Thesis.

[52] Aguirre A.A., Balazs G.H. (2000). Blood biochemistry value of green turtles, *Chelonia mydas*, with and without fibropapillomatosis. Comp. Haematol. Int..

[53] Lockau L., Atkinson S.A. (2018). Vitamin D’s role in health and disease: How does the present inform our understanding of the past?. Int. J. Paleopathol..

[54] Jakobsen J., Jensen S.K., Hymøller L., Anderson E.W., Kaas P., Burild A., Jäpelt R.B. (2015). *Short communication*: Artificial ultraviolet B light exposure increases vitamin D levels in cow plasma and milk. J. Dairy Sci..

[55] Ferguson G.W., Gehrmann W.H., Hammack S.H., Chen T.C., Holick M.F., Holick M.F. (2002). Effects of dietary vitamin D and UV-B exposure on voluntary exposure to ultraviolet light, growth and survival of the panther chameleon. Biologic Effects of Light 2001.

[56] Karsten K.B., Ferguson G.W., Chen T.C., Holick M.E. (2009). Panther chameleons, *Furcifer pardalis*, behaviorally regulate optimal exposure to UV depending on dietary vitamin D_3_ status. Physiol. Biochem. Zool..

[57] Donoghue S., Mader D.R. (2006). Nutrition. Reptile Medicine and Surgery.

[58] Flint M., Morton J.M., Limpus C.J., Patterson-Kane J.C., Murray P.J., Mills P.C. (2010). Development and application of biochemical and haematological reference intervals to identify unhealthy green sea turtles (*Chelonia mydas*). Vet. J..

[59] Nussey S., Whitehead S. (2001). Endocrinology: An Integrated Approach.

[60] Leary P.F., Zamfirova I., Au J., McCracken W.H. (2017). Effect of Latitude on Vitamin D Levels. J. Am. Osteopath. Assoc..

[61] Southworth L.O., Holick M.F., Chen T.C., Kunz T.H. (2013). Effects of sunlight on behavior and 25-hydroxyvitamin D levels in two species of old world fruit bats. Dermatoendocrinology.

[62] Duffy D.J., Schnitzler C., Karpinski L., Thomas R., Whilde J., Eastman C., Yang C., Krstic A., Rollinson D., Zirkelbach B. (2018). Sea turtle fibopapilloma tumors share genomic drivers and therapeutic vulnerabilities with human cancers. Commun. Biol..

[63] Musick J.A., Limpus C.J., Lutz P.L., Musick J.A. (1997). Habitat utilization and migratino in juvenile sea turtles. The Biology of Sea Turtles.

[64] Häder D.P., Williamson C.E., Wängberg S.Å., Rautio M., Rose K.C., Gao K., Helbling E.W., Sinha R.P., Worrest R. (2015). Effects of UV radiation on aquatic ecosystems and interactions with other environmental factors. Photochem. Photobiol. Sci..

[65] Laing C.J., Fraser D.R. (1999). The vitamin D system in iguanian lizards. Comp. Biochem. Physiol. B Biochem..

[66] Perrault J.R., Levin M., Mott C.R., Bovery C.M., Bresette M.J., Chabot R.M., Gregory C.R., Guertin J.R., Hirsch S.E., Ritchie B.W. (2021). Insights on immune function in free-ranging green sea turtles (*Chelonia mydas*) with and without fibropapillomatosis. Animals.

[67] Rousselet E., Stacy N.I., LaVictoire K., Higgins B.M., Tocidlowski M.E., Flanagan J.P., Godard-Codding C.A.J. (2013). Hematology and plasma biochemistry analytes in five age groups of immature, captive-reared loggerhead sea turtles (*Caretta caretta*). J. Zoo Wildl. Med..

